# iP3T: an interpretable multimodal time-series model for enhanced gait phase prediction in wearable exoskeletons

**DOI:** 10.3389/fnins.2024.1457623

**Published:** 2024-09-04

**Authors:** Hui Chen, Xiangyang Wang, Yang Xiao, Beixian Wu, Zhuo Wang, Yao Liu, Peiyi Wang, Chunjie Chen, Xinyu Wu

**Affiliations:** ^1^ShenZhen College of Advanced Technology, University of Chinese Academy of Sciences, Shenzhen, China; ^2^Chinese Academy of Sciences Key Laboratory of Human-Machine-Intelligence Synergic Systems, Shenzhen Institutes of Advanced Technology, Chinese Academy of Sciences, Shenzhen, China; ^3^Guangdong Provincial Key Lab of Robotics and Intelligent System, Shenzhen Institutes of Advanced Technology, Chinese Academy of Sciences, Shenzhen, China; ^4^Department of Mechanical Engineering, National University of Singapore, Singapore, Singapore

**Keywords:** multimodal data fusion, gait phase prediction, wearable exoskeleton, transformer, IMU, sEMG, stretch sensors

## Abstract

**Introduction:**

Wearable exoskeletons assist individuals with mobility impairments, enhancing their gait and quality of life. This study presents the iP3T model, designed to optimize gait phase prediction through the fusion of multimodal time-series data.

**Methods:**

The iP3T model integrates data from stretch sensors, inertial measurement units (IMUs), and surface electromyography (sEMG) to capture comprehensive biomechanical and neuromuscular signals. The model's architecture leverages transformer-based attention mechanisms to prioritize crucial data points. A series of experiments were conducted on a treadmill with five participants to validate the model's performance.

**Results:**

The iP3T model consistently outperformed traditional single-modality approaches. In the post-stance phase, the model achieved an RMSE of 1.073 and an R^2^ of 0.985. The integration of multimodal data enhanced prediction accuracy and reduced metabolic cost during assisted treadmill walking.

**Discussion:**

The study highlights the critical role of each sensor type in providing a holistic understanding of the gait cycle. The attention mechanisms within the iP3T model contribute to its interpretability, allowing for effective optimization of sensor configurations and ultimately improving mobility and quality of life for individuals with gait impairments.

## 1 Introduction

With the rapid progression of global aging, the proportion of the elderly population has significantly increased, leading to a notable decline in mobility and a rise in gait disorders among older adults. Additionally, the number of patients with hemiplegia and other motor disorders, caused by conditions such as stroke, spinal cord injuries, and multiple sclerosis, is also growing annually (Hobbs and Artemiadis, 2020; Murray et al., 2014; Huang and Krakauer, 2009). These elderly individuals and patients require long-term rehabilitation and assistive devices to enhance their quality of life (Morawietz and Moffat, 2013). In this context, lower limb powered exoskeletons, as an emerging rehabilitation assistive device, have gradually demonstrated their importance (Schwartz and Meiner, 2015; Ding et al., 2005). A lower limb powered exoskeleton is a wearable device that assists or enhances lower limb movement through mechanical and electronic control systems (Yan et al., 2015).

As illustrated in **Figure 2**, gait phase prediction is essential for providing active assistance with lower limb exoskeletons during walking. Human walking is typically a cyclical motion, where the interval between successive occurrences of the same gait event is termed a gait cycle (Ren et al., 2007). Gait events include toe off, initial contact, and heel off. Continuous gait phase prediction divides the cyclical motion into time steps ranging from 0 to 100%, while discrete gait cycle prediction segments the gait cycle into different phases based on various movement states. This work delineates four gait phases: pre-stance (0–30%), post-stance (31–60%), pre-swing (61–80%), and post-swing (81–100%).

In recent years, many gait phase prediction methods have utilized one or more wearable sensors to capture kinematic information about human walking and employed deep learning methods to predict gait cycles. These sensors include IMUs, sEMG, angle sensors, and force-sensitive resistors (FSRs; Su et al., 2020; Wang et al., 2022b; Luo et al., 2019; Wu et al., 2021; Malcolm et al., 2013; Mohr et al., 2019; Suo et al., 2010; Heo et al., 2015; Wang et al., 2023a,b, 2022a, 2020). However, many of these models lack interpretability and struggle with the computational complexity of high-dimensional data. The iP3T model leverages the strengths of multimodal data fusion and transformer architectures to address these limitations, providing more accurate and interpretable predictions. These approaches enable more accurate and real-time gait phase prediction, thus effectively controlling exoskeleton devices to provide better assistance to users. Wu et al. proposed a model based on Graph Convolutional Networks (GCNs) for gait phase classification, using goniometers and FSRs to control lower limb exoskeletons (Wu et al., 2021). This model can identify four phases of one leg gait during walking: heel strike, foot flat, heel off, and swing, achieving a maximum gait phase classification accuracy of 97.43%. However, as the input data dimension increases, the computational complexity of Graph Neural Networks grows linearly, putting pressure on edge computing devices. Wang et al. developed a neural network algorithm based on multimodal information fusion (a two-layer linear feedforward neural network) for gait phase prediction, employing IMUs and FSRs (Wang et al., 2023b). This method achieved satisfactory gait phase prediction performance; however, the linear layer deep learning model makes it challenging to understand how the model operates, and adding sensors or increasing channels is difficult. Luo et al. proposed a low-cost yet effective end-to-end gait subphase recognition system based on sEMG (Luo et al., 2019). The system comprises a wireless multi-channel signal acquisition device that simultaneously collects thigh muscle sEMG and foot pressure signals, and a novel neural network sEMG signal classifier combining Long Short-Term Memory (LSTM) networks (Ding et al., 2018) and Multilayer Perceptrons (MLPs). This system's average recognition accuracy is significantly higher than that of other classic methods (SVM; Li et al., 2016, KNN; Kim et al., 2011, and LDA; Joshi et al., 2013). However, traditional and LSTM structures rely on sequential processing with fixed time steps, preventing them from directly attending to any position within the input sequence.

Different sensors can capture various dimensions of motion signals; for example, foot pressure signals detect footfall timing, EMG signals contain muscle contraction information, while IMUs and angle sensors capture joint angles, angular velocity, and angular acceleration. The fusion of multiple sensors allows for a comprehensive analysis of human motion (Nweke et al., 2019). However, due to the computational power limitations of edge devices, large datasets are challenging for deep learning models to fit and can reduce the speed of forward computations (Chen and Ran, 2019). Simply stacking multiple sensors and channels does not necessarily improve the accuracy of downstream tasks like gait phase prediction and may compromise real-time performance. Traditional deep learning methods, such as CNNs, LSTMs, and GRUs (Su et al., 2019; Bruinsma and Carloni, 2021), function as black boxes, making it difficult to understand their forward computations and optimize sensor configurations.

With the popularity of multimodal general models, Transformers have been widely applied as backbone networks in various tasks (Nagrani et al., 2021). The core of the Transformer model is the self-attention mechanism, which can capture dependencies between different parts of the input sequence. Attention weights provide a clear understanding of which parts of the input the model focuses on during processing. This interpretability aids researchers and users in understanding the model's decision-making process, helping to optimize sensor configurations (Vaswani et al., 2017).

In this study, we propose an interpretable Patch Time-series Token Transformer (iP3T) model based on the patch time series Transformer (PatchTST, Nie et al., 2022) architecture, predicting continuous gait cycle changes from multiple sensors, including multi-channel sEMG sensors, angle stretch sensors, IMUs, and foot pressure signals. The model can determine the contribution weights of different time-series sequences to gait phase prediction, allowing for the selection of effective gait sequences and optimization of sensor channel configurations. It also demonstrates the attention scope of the model within specific channels. Additionally, its performance not only surpasses several classic time-series prediction models, including CNNs, LSTMs, GRUs, and their combinations (Su et al., 2019; Bruinsma and Carloni, 2021), but it can also enhance the gait phase prediction performance of these traditional models by optimizing sensor channels. This suggests that even with the emergence of more powerful backbone networks in the future, the proposed iP3T can still be utilized to further improve the accuracy of gait phase prediction.

## 2 Methods

### 2.1 Gait data acquisition

#### 2.1.1 Participant recruitment

**Figure 1:**
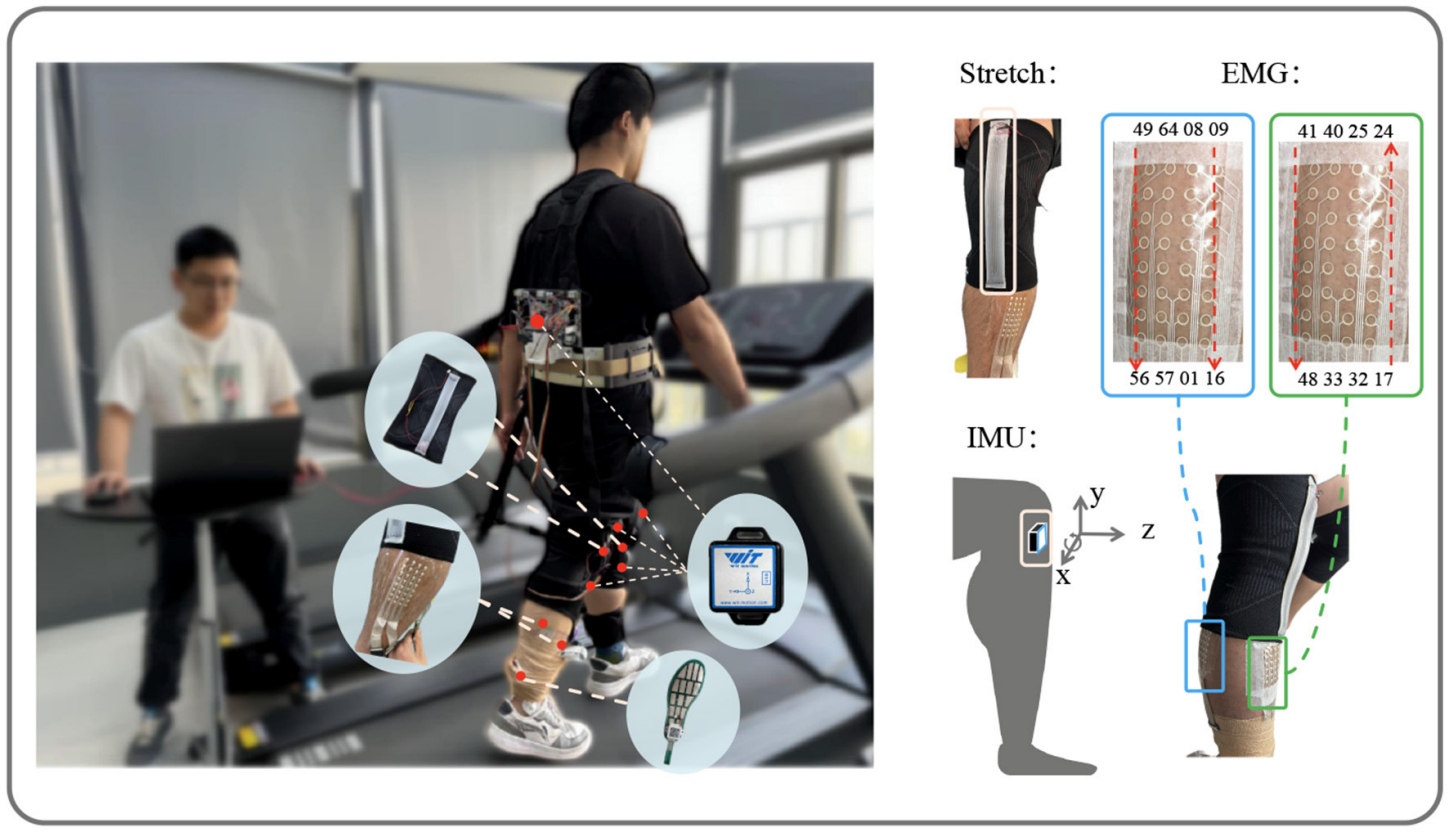
Data acquisition. Showing the setup with stretch, IMU, and EMG sensors on the subject. The stretch sensors and EMG channel positions are marked, along with the IMU's axis directions (x, y, z) for data collection.

As illustrated in Figure 1, to conduct gait prediction and validate the method's effectiveness, we recruited five non-disabled participants for the experiment. The data of the participants are shown in the Table 1. The study was approved by the Institutional Review Board of the Shenzhen Institutes of Advanced Technology, Chinese Academy of Sciences, in compliance with ethical standards (Approval No. SIATIRB-231115-H0681). All participants provided informed consent before the experiment and were informed that they could withdraw from the study at any time.

**Table 1:** Participant data statistics.

**Participant ID**	**Gender**	**Age**	**Height (cm)**	**Weight (kg)**
1	Male	28	170	51
2	Female	28	172	65
3	Male	24	173	70
4	Female	23	162	55
5	Male	31	177	81

#### 2.1.2 Sensor settings

Two stretch sensors, as described in Xiao et al. (2023), with a sampling frequency of 100 Hz, were worn on the knee joints of both legs to collect angle variation information. The multi-channel sEMG sensors, as described in Yang et al. (2023), comprised two patches, each with a sampling frequency of 2,000 Hz. One patch, covering channels 17–48, was placed on the tibialis anterior of the right leg, while the other patch, covering channels 1–16 and 49–64, was placed on the right gastrocnemius. To synchronize the input data, the multi-channel sEMG data were downsampled to 100 Hz. Five IMUs(Witmotion, China), each with a sampling frequency of 100 Hz, were attached to the left thigh, right thigh, left shank, right shank, and trunk, all oriented with the z-axis pointing in the walking direction. Each IMU recorded angles, angular velocities, and angular accelerations along the x, y, and z axes.

**Figure 2:**
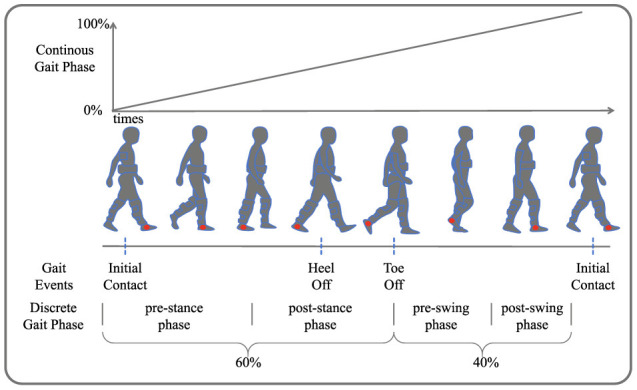
Gait phase segmentation. Four gait events are depicted: initial contact, heel off, toe off, and another initial contact. The continuous gait phase prediction ranges from 0 to 100%, illustrating the transition through pre-stance, post-stance, pre-swing, and post-swing phases.

In total, the stretch sensors contributed two channels, the sEMG sensors provided 64 channels, and the IMUs added 5 × 3 × 3 channels. The distributed foot pressure data, used as the label, were averaged across channels to form a single channel. The foot pressure time-series data were smoothed using a moving average filter, with local maxima marked. Linear interpolation between all marked points was used to scale the data from 0 to 100. The gait phase segmentation results, shown in Figure 2, use the initial contact event of the right foot as the starting point for continuous gait data segmentation. The data processing process can be referred to Section 2.3.1.

#### 2.1.3 Data acquisition

After a warm-up session, participants walked on a treadmill at a speed of 4.5 km/h with the following sensor deployment: stretch sensors, multi-channel distributed sEMG sensors, IMUs, and distributed foot pressure sensors. The time-series data were meticulously synchronized and transmitted to a laptop via a data acquisition board. The first three sensors provided input data for the model, while the foot pressure data served as the label.

### 2.2 iP3T model architecture

**Figure 3:**
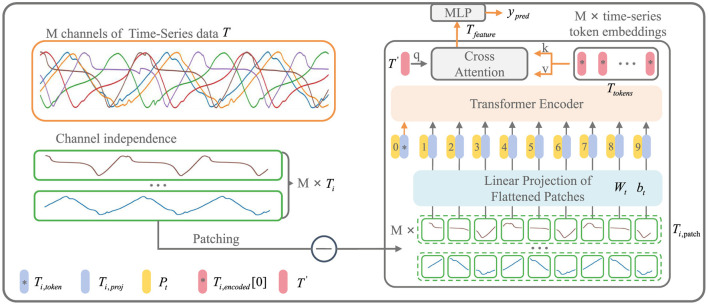
iP3T model architecture. The iP3T model processes M channels of time-series data, denoted as **T**, with each channel **T***i* independently divided into patches. Each patch is linearly projected into the embedding space using parameters **W**t and **b**t. The patches, along with a learnable global token **T***i*, token, are encoded with positional information **P**t and fed into the Transformer encoder. The attention mechanism in the encoder assigns importance to different segments of the time-series data, with cross-attention refining the predictions. The model outputs predictions **y**_pred_ through a Multi-Layer Perceptron (MLP). The symbols at the bottom left mark the key components in the model.

As shown in Figure 3, the input to the iP3T model is time-series data from different sensors obtained through a sliding window approach, where the total number of channels is *M* and the time steps are *L*. The time series data is represented as **T** ∈ ℝ^*M* × *L*^


(1) $$\begin{array}{c} \begin{array}{c} {\text{T}}_{i,\text{patch}}=\text{Patch}\left({\text{T}}_{i}\right)\in {\mathbb{R}}^{\left(L/P_{l}\right)\times P_{l}}\\ {\text{T}}_{i,\text{proj}}={\text{T}}_{i,\text{patch}}{\text{W}}_{\text{t}}+{\text{b}}_{\text{t}}\\ {\text{T}}_{i,\text{embed}}=\left[{\text{T}}_{i,\text{token}};{\text{T}}_{i,\text{proj}}\right]\in {\mathbb{R}}^{\left(L/P_{l}+1\right)\times d}\\ {\text{T}}_{i,\text{input}}={\text{T}}_{i,\text{embed}}+{\text{P}}_{\text{t}} \end{array} \end{array}$$

where for *i* = 1, 2, …, *M*, ${\text{W}}_{\text{t}}\in {\mathbb{R}}^{P_{l}\times d}$, ${\text{b}}_{\text{t}}\in {\mathbb{R}}^{1\times d}$, **T**_*i*,token_ is a Learnable Token ∈ ℝ^1×*d*^, ${\text{P}}_{\text{t}}\in {\mathbb{R}}^{\left(L/P_{l}+1\right)\times d}$.

Operating independently of the channels, **T**_*i*_ represents a single channel time-series sequence among the *M* channels. The patch operation effectively balances the preservation of the sequential order and local information of the time series data. Each **T**_*i*_ is divided into *L*/*P*_*l*_ patches of length *P*_*l*_ through the Patch operation. The parameters **W**_t_ and **b**_t_ represent a learnable linear mapping layer that projects the flattened time-series patches into the embedding space, mapping the time-series information into *d*-dimensional vectors. A learnable global token **T**_*i*,token_ is then used to aggregate and summarize the time-series information from each patch through various attention weight distributions (Devlin et al., 2018). The positional information of the global token and each patch within the original sequence is encoded by **P**_t_, forming the input sequence to the Transformer encoder (Vaswani et al., 2017).


(2) $$\begin{array}{c} \begin{array}{c} {\text{T}}_{i,\text{encoded}}={\text{Encoder}}_{t}\left({\text{T}}_{i,\text{input}}\right)\\ {\text{T}}_{\text{tokens}}=\left[{\text{T}}_{1,\text{encoded}}\left[0\right];{\text{T}}_{2,\text{encoded}}\left[0\right];\ldots ;{\text{T}}_{M,\text{encoded}}\left[0\right]\right]\\ {\text{T}}_{\text{feature}}=\text{CrossAttention}\left({\text{T}}^{\prime }W_{Q},{\text{T}}_{\text{tokens}}W_{K},{\text{T}}_{\text{tokens}}W_{V}\right)\\ {ŷ}_{\text{pred}}={\text{MLP}}_{\text{pred}}\left({\text{T}}_{\text{feature}}\right) \end{array} \end{array}$$

where *W*_*Q*_, *W*_*K*_, *W*_*V*_ are the projection matrices for the query, key, and value, ${\text{T}}_{\text{tokens}}\in {\mathbb{R}}^{M\times d}$, and **T′** is a Learnable Token ∈ ℝ^1×*d*^. Both cross-attention and attention mechanisms utilize dot-product attention.

**T**_*i*,encoded_ represents the encoded result from the Transformer encoder. From each of the *M* channels, the **T**_*i*,token_ is extracted to form **T**_tokens_. A learnable global time-series token, **T′**, is then used in CrossAttention to aggregate the characteristic information from all the time-series channels. The resulting **T**_feature_ is processed through an MLP layer to obtain the prediction result, ŷ_pred_, which is a two-dimensional vector ∈ ℝ^2^.

**Algorithm 1:** Interpretable P3T encoder.

**Input:** Time series data **T**
**Output:** Predicted values ŷ_pred_
1: Patch, project, and encode the time series data for each channel:
2: **for** each channel *i* **do**
3: **T**_*i*,patch_ ← Patch(**T**_*i*_), **T**_*i*,proj_ ← **T**_*i*,patch_**W**_t_ + **b**_t_, **T**_*i*,embed_ ← [**T**_*i*,token_; **T**_*i*,proj_], **T**_*i*,input_ ← **T**_*i*,embed_ + **P**_t_, **T**_*i*,encoded_ ← Encoder_*t*_(**T**_*i*,input_)
4: **end for**
5: Collect the encoded time series tokens: **T**_tokens_ ← [**T**_1,encoded_[0], **T**_2,encoded_[0], …, **T**_*M*,encoded_[0]]
6: Apply cross attention: ${\text{T}}_{\text{feature}}\leftarrow \text{CrossAttention}\left({\text{T}}^{\prime },{\text{T}}_{\text{tokens}},{\text{T}}_{\text{tokens}}\right)$
7: Make predictions: ŷ_pred_ ← MLP_pred_(**T**_feature_)
8: **return** ŷ_pred_

This prediction is then converted back to the gait cycle using the following formula:


(3) $$\begin{array}{c} \begin{array}{c} \theta =\text{atan2}\left({ŷ}_{\text{pred}}\left[0\right],{ŷ}_{\text{pred}}\left[1\right]\right)\\ \text{if }\theta <0,\text{then }\theta =\theta +2\pi \\ \text{gait phase}=\frac{\theta \cdot 100}{2\pi } \end{array} \end{array}$$

In the Encoder_*t*_, self-attention is utilized to capture dependencies within the input sequence. Multi-head attention combines multiple attention heads to enhance the model's capacity to focus on different parts of the input. The self-attention output is then added to the input and normalized. Following this, a feed-forward network is applied to the normalized self-attention output, and the result is again added and normalized. This sequence of operations constitutes a single layer of the Transformer encoder, which is repeated for *N* layers:


(4) $$\begin{array}{c} \begin{array}{c} \text{Attention}\left(Q^{\prime },K^{\prime },V^{\prime }\right)=\text{softmax}\left(\frac{Q^{\prime }{K\prime }^{T}}{\sqrt{d_{k}}}\right)V^{\prime }\\ {\text{head}}_{j}=\text{Attention}\left(QW_{Q_{j}},KW_{K_{j}},VW_{V_{j}}\right)\\ \text{MultiHead}\left(Q,K,V\right)=\text{Concat}\left({\text{head}}_{1},{\text{head}}_{2},\ldots ,{\text{head}}_{h}\right)W_{O}\\ {\text{T}}_{i,\text{attention}}=\text{LayerNorm}\left({\text{T}}_{i,\text{input}}+\text{MultiHead}\left(Q,K,V\right)\right)\\ \text{FFN}\left(x\right)=\text{GeLU}\left(xW_{1}+b_{1}\right)W_{2}+b_{2}\\ {\text{T}}_{i,\text{encoded}}=\text{LayerNorm}\left({\text{T}}_{i,\text{attention}}+\text{FFN}\left({\text{T}}_{i,\text{attention}}\right)\right) \end{array} \end{array}$$

where *Q* = **T**_*i*,input_, *K* = **T**_*i*,input_, *V* = **T**_*i*,input_ in the *Attention*, *W*_*Q*_*j*__, *W*_*K*_*j*__, *W*_*V*_*j*__ are the projection matrices for each head, for*j* = 1, …, *h, d*_*k*_ = *d*. *W*_*O*_ is the output projection matrix. The Gaussian Error Linear Unit (GELU) is an activation function that utilizes a Gaussian distribution for activation, effectively handling non-linear and complex features. Layer Normalization (LayerNorm) is a normalization technique that standardizes each layer's neurons to have zero mean and unit variance, thereby enhancing the training stability and convergence speed of the model.

### 2.3 Experiment settings

#### 2.3.1 Gait data processing

The collected multi-modal time-series data from Section 1 underwent channel selection. The foot pressure sensor averaged the distributed data into a single channel to serve as the label for gait phase segmentation. For the IMU data, we selected 16 channels: the x-axis angle, angular velocity, and angular acceleration for both the left and right thighs; the x-axis angle, angular velocity, and angular acceleration, as well as the z-axis acceleration, for the left and right shanks; and the x-axis angle, angular velocity, and angular acceleration for the trunk. These channels were labeled as follows: L_T_agl_x (left thigh x-axis angle), L_T_gyro_x (left thigh x-axis angular velocity), L_T_acc_x (left thigh x-axis angular acceleration), R_T_agl_x (right thigh x-axis angle), R_T_gyro_x (right thigh x-axis angular velocity), R_T_acc_x (right thigh x-axis angular acceleration), L_S_agl_x (left shank x-axis angle), L_S_gyro_x (left shank x-axis angular velocity), L_S_acc_x (left shank x-axis angular acceleration), L_S_acc_z (left shank z-axis acceleration), R_S_gyro_x (right shank x-axis angular velocity), R_S_acc_x (right shank x-axis angular acceleration), R_S_acc_z (right shank z-axis acceleration), Tk_agl_x (trunk x-axis angle), Tk_gyro_x (trunk x-axis angular velocity), and Tk_acc_x (trunk x-axis angular acceleration).

For the stretch sensors, we collected signals from the left and right legs, labeled as L_leg_str (left leg stretch) and R_leg_str (right leg stretch), comprising two channels.

For the sEMG, we selected 16 channels from the 64 available channels, with every fourth channel being chosen: EMG_1, EMG_5, EMG_9, EMG_13, EMG_17, EMG_21, EMG_25, EMG_29, EMG_33, EMG_37, EMG_41, EMG_45, EMG_49, EMG_53, EMG_57, and EMG_61. Channels 17, 21, 25, 29, 33, 37, 41, and 45 captured signals from the tibialis anterior muscle, while the others captured signals from the gastrocnemius muscle.

The foot pressure sensor averaged the distributed data into a single channel to serve as the label for gait phase segmentation.

To enable the model to perceive contextual information over a longer time span, we applied a sliding window of size *M* × 100 time steps, with *M* being the total number of channels and a stride of one time step, across the input data sources (IMU, Stretch, sEMG). This enriched our dataset, resulting in each sample having a size of *M* × 100.

The value range of the gait phase, obtained from local maxima points in the foot pressure data through linear interpolation, was 0–100, which was then converted to label values using the formula (Kang et al., 2019):


(5) $$\begin{array}{c} \begin{array}{c} {\text{y}}_{\text{label[0]}}=\sin\left(\text{gait phase}\times 2\pi /100\right)\\ {\text{y}}_{\text{label[1]}}=\cos\left(\text{gait phase}\times 2\pi /100\right) \end{array} \end{array}$$

This conversion prevented the model from yielding inaccurate predictions near boundary values, as the kinematic information at 0 and 100% is nearly identical.

#### 2.3.2 Training and evaluation performance metric

After processing with the sliding window, our dataset comprised 16,000 samples, which were split into training and testing sets at a 7:3 ratio. After training the iP3T model on the IMU, Stretch, and sEMG data sources, we analyzed the cross-attention weights of *T*′.


(6) $$\begin{array}{c} \begin{array}{c} \text{Weights}=\text{Softmax}\left(\frac{QK^{T}}{\sqrt{d_{k}}}\right) \end{array} \end{array}$$

where $Q={\text{T}}^{\prime }W_{Q}$ and *K* = **T**_tokens_*W*_*K*_

Based on these weights, we selected the top 8 prioritized time-series channels from EMG and IMU to form a 16-channel multi-modal data source (IMU+EMG). Similarly, we selected the top 14 channels from EMG and IMU and added 2 channels from the stretch sensors to form the 16-channel multi-modal data sources (Stretch+EMG, IMU+Stretch). Finally, we selected the top 7 channels from IMU and EMG and added 2 channels from the stretch sensors to form the 16-channel multi-modal data source (IMU+Stretch+EMG).

We then trained the iP3T model on these new data sources and validated the performance improvements in gait phase prediction. Additionally, we used classical time-series prediction methods, including CNN, LSTM, GRU, CNN+LSTM, and CNN+GRU, as baselines (Su et al., 2019; Bruinsma and Carloni, 2021). These models were trained on the multi-modal data sources (IMU+Stretch+EMG) and compared against the proposed iP3T model for performance metrics.

Training was conducted on a server with the following specifications: CPU: 12th Gen Intel(R) Core(TM) i7-12700, GPU: NVIDIA GeForce RTX 3080Ti, RAM: 32GB. The software environment included Python 3.8, Pytorch version 1.11.0+cu115, CUDA Version 11.5, and cuDNN version 8302. The Adaptive Moment Estimation (Adam) optimizer was used, with a batch size of 32 and a learning rate of 4e-6. Training ran for 200 epochs, with RMSE and R2 metrics used to evaluate model performance. The mean square loss (MSE loss) was employed as the loss function.

The formulas for RMSE, R2, and MSE Loss are given as follows:


(7) $$\begin{array}{c} \begin{array}{c} \text{RMSE}=\sqrt{\frac{1}{n}\sum_{i=1}^{n}{\left(y_{i}-{ŷ}_{i}\right)}^{2}} \end{array} \end{array}$$

(8) $$\begin{array}{c} \begin{array}{c} \text{R2}=1-\frac{\sum_{i=1}^{n}{\left(y_{i}-{ŷ}_{i}\right)}^{2}}{\sum_{i=1}^{n}{\left(y_{i}-ȳ\right)}^{2}} \end{array} \end{array}$$

(9) $$\begin{array}{c} \begin{array}{c} \text{MSE Loss}=\frac{1}{n}\sum_{i=1}^{n}{\left(y_{{\text{pred}}_{i}}-y_{{\text{label}}_{i}}\right)}^{2} \end{array} \end{array}$$

In the iP3T model, the patch size(*P*_*l*_) was set to 10 with no overlap between patches. The vector dimension *d* was set to 768, with 12 attention heads and 6 layers. The FNN used *W*1 to expand the *d*-dimensional vector to 4*d* and *W*2 to reduce it back to *d*. For the CNN baseline, we aligned the input channel with our dataset while maintaining the dual-channel convolutional neural network architecture as implemented by Su et al. (2019). The LSTM, GRU, CNN+LSTM, and CNN+GRU models were implemented according to the configurations in Julia's paper (Bruinsma and Carloni, 2021), with the hidden layer dimensions for LSTM and GRU set to 128, each having four RNN blocks. The CNN+LSTM and CNN+GRU models consisted of three CNN blocks connected to three LSTM or GRU blocks, with input dimensions adjusted accordingly.

### 2.4 Exoskeleton platform setting

**Figure 4:**
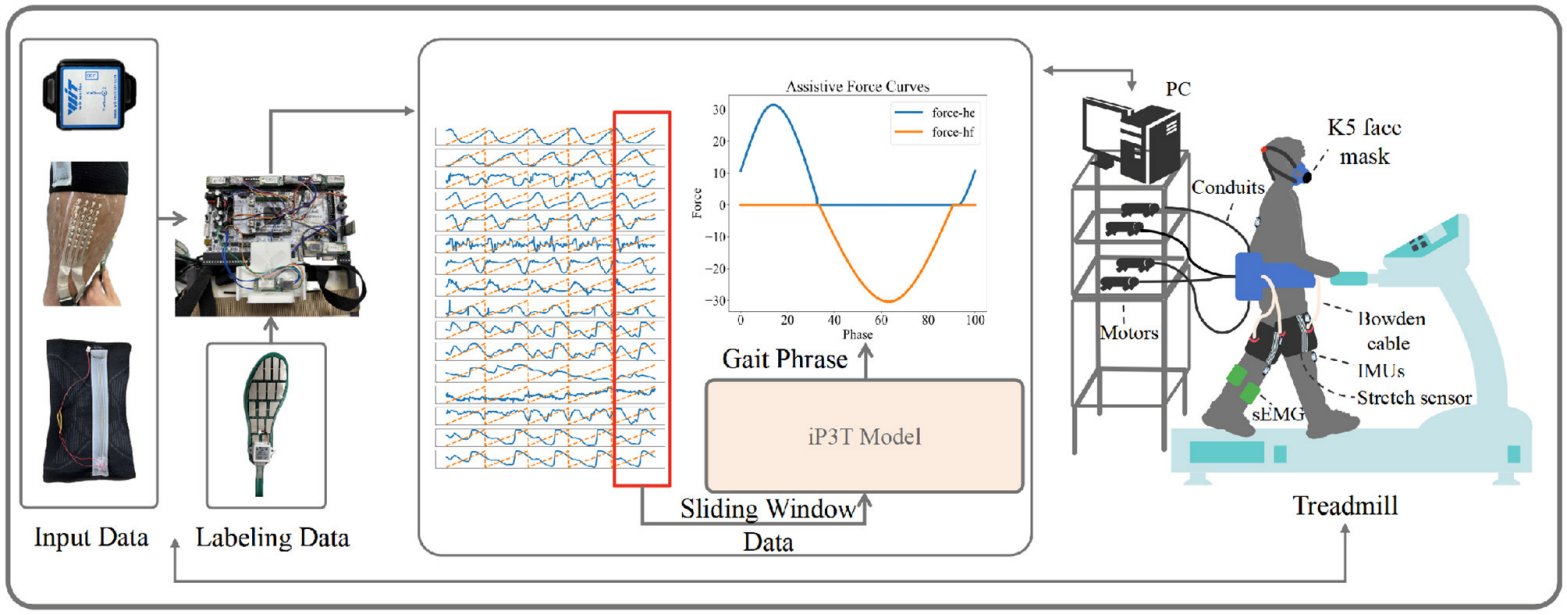
The experimental setup for validating the iP3T model is depicted. Input data from IMU, Stretch, and sEMG sensors are collected and transmitted to a computer via a data acquisition board. A FIFO queue captures real-time multimodal linear time-series data using a sliding window. The iP3T model predicts the gait phase, informing assistive force curves (force-he and force-hf) that drive motors attached to Bowden cables, providing hip assistance. The setup includes a K5 face mask for metabolic measurement, showcasing the integration of sensors and the assistive treadmill to enhance gait assistance.

Finally, we validated the enhanced performance of the iP3T model's gait phase predictions achieved through multimodal data fusion by comparing the exoskeleton assistance on the treadmill with that based on single-modality stretch predictions. The COSMED K5, a wearable metabolic system, was used for this validation. As shown in Figure 4, after equipping the recruited subjects with Stretch, IMU, and EMG sensors, metabolic consumption experiments were conducted on a treadmill at speeds of 3.2, 4.3, and 5.4 km/h. Data were transmitted to a computer in real-time via a data acquisition board. We employed a FIFO queue to implement a sliding window, enabling real-time collection of multimodal linear time-series data, which were then fed into the iP3T model deployed on the computer. Our platform's flexible exoskeleton for the assistive treadmill, consisting of four motors and four Bowden cables attached at the knee joints, provided hip assistance. On the assistive treadmill, predictions were made using the iP3T model trained on Stretch and IMU+Stretch+EMG data, yielding continuous gait cycles. The assistance was based on force mapping functions (The hip assistance curves used in this study are derived from Bryan's work, Bryan et al., 2021), where positive values represent clockwise pulling to assist hip extension (force-he) and negative values represent counterclockwise pulling to assist hip flexion (force-hf). These force mapping functions drove the motors to pull the Bowden cables accordingly, providing the necessary support based on the predicted gait cycles.

## 3 Results and discussion

### 3.1 Predictions from different data sources in the iP3T model

**Figure 5:**
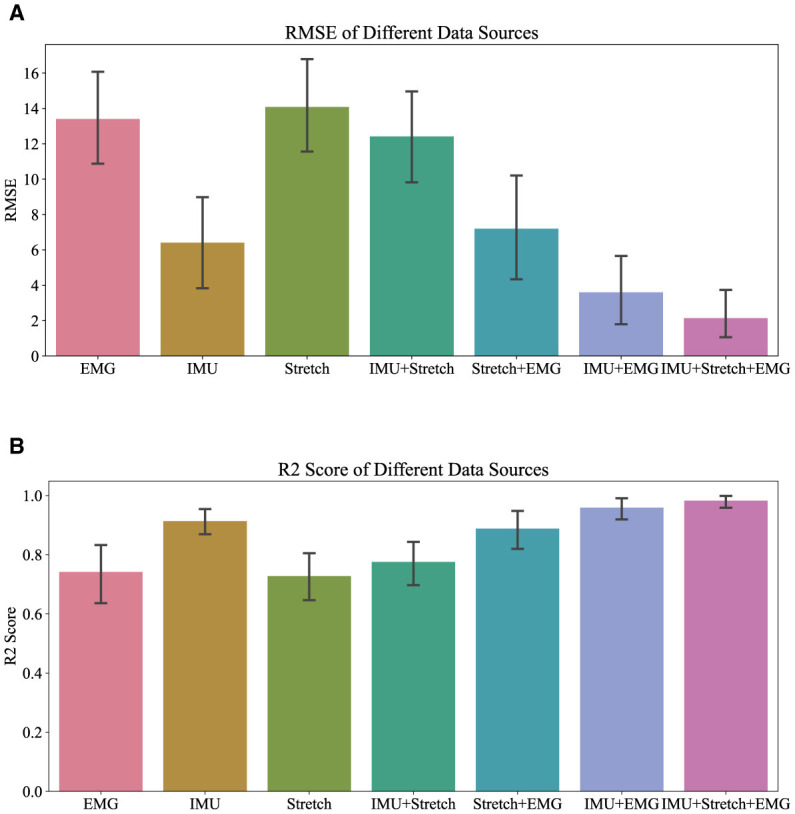
Overall R2 and RMSE performance of iP3T on different data sources.

**Figure 6:**
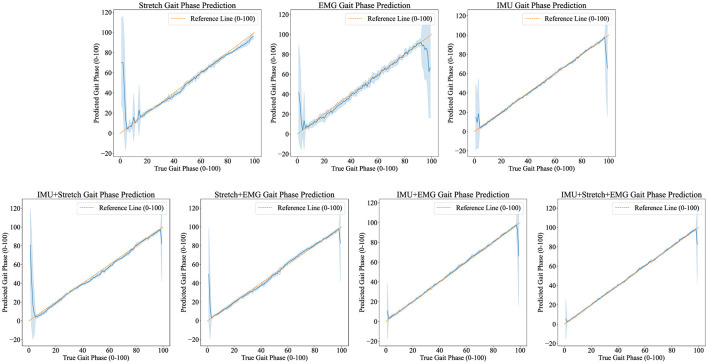
Predicted results visualization displays the iP3T model's gait phase prediction results using various data sources: Stretch, EMG, IMU, IMU+Stretch, Stretch+EMG, IMU+EMG, and IMU+Stretch+EMG. Blue solid lines represent the mean predictions, while shaded areas indicate the standard deviation. The orange dashed reference line marks the ideal prediction range (0–100).

Figures 5, 6 demonstrate the performance of the iP3T model across seven different data sources: Stretch, sEMG, IMU, IMU+Stretch, Stretch+sEMG, IMU+sEMG, and IMU+Stretch+sEMG. In Figure 5, the bar charts represent the mean values, with error bars indicating the 95% confidence intervals. In Figure 6, the blue solid line represents the mean predicted values, while the shaded area indicates the standard deviation. This visual representation clearly illustrates that multimodal time-series data fusion enhances gait phase prediction performance. However, we observe boundary effects at the edges (0 and 100) across all data sources, where the prediction accuracy deteriorates. This occurs because, to improve the model's generalization, we mapped the gait phase to Cartesian coordinates. Directly using Cartesian coordinates with MSE loss could lead to larger losses near the boundaries (0 and 100), but mapping to Cartesian coordinates minimizes this issue. Consequently, when mapped back, the prediction variance near the boundaries may be larger.

For the Stretch sensor data, the consistency between predicted and actual values is relatively good, although there is notable variability, especially at the start of the gait cycle (0–20%). These regions exhibit higher standard deviations and greater fluctuations, indicating lower reliability in these phases. However, there is no significant boundary effect in the late swing phase. Similarly, the sEMG data shows reasonable consistency with the actual values but displays greater variability in the mid-gait cycle, making it the data source with the highest standard deviation among the three single data sources. In contrast, the IMU data predictions closely match the actual values with minimal deviation throughout the gait cycle. The IMU data has the smallest standard deviation compared to other single sensor data sources, indicating more reliable predictions.

Combining data sources improves performance. For instance, the IMU+Stretch sensor combination slightly enhances prediction accuracy and reduces variability compared to using the Stretch sensor alone, as evidenced by the reduced standard deviation. Similarly, the Stretch+sEMG combination shows significant improvements over using sEMG alone, reducing both prediction error and variability. The combined use of IMU and sEMG yields predictions nearly as accurate as using IMU alone but with notable error reduction, as indicated by the lower standard deviation, suggesting more robust model performance. The best performance is achieved with the combination of IMU, Stretch, and sEMG data (IMU+Stretch+sEMG). This combination produces predictions very close to the actual values throughout the gait cycle and has the smallest standard deviation among all data sources, indicating highly reliable predictions.

From the Table 2, it is evident that the iP3T model's performance varies significantly across different data sources. Single data sources have limitations; for instance, Stretch and sEMG data perform poorly in the Pre-Stance and Post-Swing phases, with high RMSE values and even negative R2 values, indicating large prediction errors and low correlation with actual data. In contrast, the IMU data source performs relatively well across all phases, especially in the Post-Stance and Pre-Swing phases, with significantly lower RMSE values and R2 values close to 1. This suggests that IMU sensors have a strong advantage in capturing the kinematic information of the gait cycle, likely due to their ability to provide detailed angle, angular velocity, and angular acceleration data, comprehensively reflecting joint and limb movements.

**Table 2:** iP3T model metric performance.

**Gait phase**	**Pre-stance**		**Post-stance**		**Pre-swing**		**Post-swing**	
**Data source**	**RMSE**	**R2**	**RMSE**	**R2**	**RMSE**	**R2**	**RMSE**	**R2**
Stretch	26.148	-8.103	2.823	0.899	2.116	0.86	**4.114**	**0.401**
EMG	17.617	-3.132	3.824	0.815	3.364	0.647	23.121	-17.931
IMU	11.06	-0.629	1.261	0.98	1.269	0.95	12.83	-4.829
IMU+Stretch	23.403	-6.292	2.504	0.921	2.251	0.842	6.013	-0.281
Stretch+EMG	16.188	-2.489	2.601	0.914	2.286	0.837	5.924	-0.243
IMU+EMG	6.521	0.434	1.272	0.98	1.57	0.923	9.964	-2.516
IMU+Stretch+EMG	**4.612**	**0.717**	**1.073**	**0.985**	**1.108**	**0.962**	5.848	-0.211

The bolded values represent the highest-performing metrics in the tables.

The advantage of multimodal data fusion lies in its ability to capture dynamic information from different dimensions of the gait cycle. For example, the angle change information provided by Stretch sensors can complement the motion data from IMU sensors, while the muscle activity signals captured by sEMG sensors provide additional information about motor control. This complementary information enhances the model's robustness and accuracy in handling complex gait cycle variations.

Combining IMU data with other sensor data sources, such as IMU+Stretch, IMU+sEMG, and IMU+Stretch+sEMG, significantly improves prediction performance. Notably, the IMU+Stretch+sEMG combination shows the lowest RMSE and highest R2 values across all gait phases, indicating that this multimodal data fusion strategy effectively leverages the strengths of each sensor and compensates for the limitations of single sensors, providing more accurate and reliable gait predictions. This suggests that multimodal data fusion strategies have important applications in gait phase prediction, offering more precise active assistance control for exoskeleton devices and enhancing the walking ability and quality of life for patients and the elderly. From these analyzes, we can conclude that the fusion of multimodal sensor data is crucial for improving gait phase prediction performance. This not only enhances prediction accuracy but also provides more reliable control signals for practical applications in exoskeleton devices, thereby more effectively assisting patients and the elderly in walking.

### 3.2 Time-series channel weights for the iP3T

**Figure 7:**
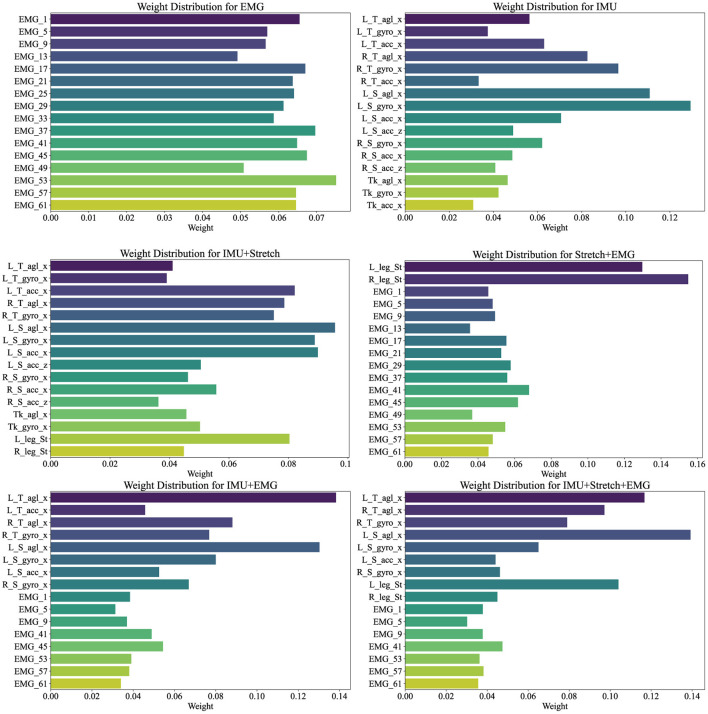
Channel weights visualization illustrates the weight distributions for various data sources (EMG, IMU, IMU+Stretch, Stretch+EMG, IMU+EMG, and IMU+Stretch+EMG) within the iP3T model. Bar lengths denote the weight magnitude, while colors distinguish different channels.

Figure 7 illustrates the weight distribution for each channel within the cross-attention mechanism of the iP3T model across various data sources: EMG, IMU, IMU+Stretch, Stretch+EMG, IMU+EMG, and IMU+Stretch+EMG. These weights are crucial as they indicate the relative importance of each channel in predicting gait phases. Analyzing these distributions reveals the contribution of different sensor modalities to model performance.

In the pure EMG data source, channels such as EMG_53, EMG_57, and EMG_61, corresponding to the gastrocnemius muscle, hold higher weights, highlighting their significant contribution to the prediction. The gastrocnemius muscle plays a crucial role in the gait cycle, especially during the stance and push-off phases, as the calf muscles need to contract strongly to propel the body forward. Electrodes on the tibialis anterior muscle, such as EMG_37, EMG_41, and EMG_45, also show relatively high weights, emphasizing their role in capturing muscle activity during the swing phase, which is essential for foot lift-off and forward swing.

In the pure IMU data source, the highest weights are attributed to the left shank's angle sensor (L_S_agl_x) and gyroscope sensor (L_S_gyro_x). This indicates the critical role of the left shank in gait phase prediction. Although the right leg's gait phase is being predicted, the left leg's data provides significant reference value. The gait cycle is a coordinated bilateral process, where the movement of one leg directly affects the other. Thus, analyzing the left shank's angle and angular velocity can provide crucial information about the entire gait cycle, aiding in predicting the right leg's gait phase. The lower weights for the trunk suggest its relatively stable motion during the gait cycle, contributing less directly to gait phase changes. The lower weights for the right leg may be due to the model effectively capturing sufficient information from the left leg to predict the right leg's gait phase, indicating a high degree of correlation between the movements of both legs.

In the IMU+Stretch data source, the left shank's sensors again show the highest weights, specifically the angle sensor (L_S_agl_x) and gyroscope sensor (L_S_gyro_x), reaffirming the left shank's crucial role. This may be attributed to the left shank sensors being positioned below the knee, allowing precise capture of angle and angular velocity changes during walking. These data provide detailed kinematic information essential for accurate gait phase prediction. The stretch sensors, despite having moderate weights, still play an important role. Positioned at the knee joint, they capture extension and flexion changes during walking, providing critical data that complement the IMU's angle and angular velocity readings. Stretch sensors offer a direct reflection of knee joint movements, aiding in refined gait phase prediction.

In the Stretch+EMG and IMU+EMG data sources, the majority of the weight is held by the Stretch and IMU sensors, reflecting their critical role in gait phase prediction. The relatively lower weights for EMG sensors may be due to their primary focus on muscle electrical activity, which, while closely related to movement control, may not be as directly impactful for specific gait phase prediction as kinematic data from angle and velocity sensors. The high-frequency sampling of EMG provides detailed muscle activity information, but its effectiveness in gait prediction might not be as straightforward as motion sensor data. Additionally, EMG signals are susceptible to noise, such as sweating during exercise, potentially affecting signal stability and resulting in lower model weights.

For the IMU+Stretch+EMG data source, despite the lower weights for EMG sensors, the combined use of IMU, Stretch, and EMG sensors outperforms the IMU+Stretch sensor combination, as indicated in Table 2. This highlights that integrating data from various sensors enables the model to capture subtle variations in the gait cycle more comprehensively, enhancing prediction accuracy and robustness. While EMG sensors have lower weights, they still provide valuable supplementary information during specific gait phases.

The analysis of cross-attention weights across different data sources in the iP3T model emphasizes the importance of multimodal data fusion. Each sensor type whether capturing muscle activity, joint angles, or limb dynamics adds a unique layer of information crucial for comprehensive gait phase prediction. The balanced weight distribution in multimodal configurations, such as IMU+Stretch+EMG, underscores the complementary nature of these data sources. EMG sensors offer detailed muscle activity data essential for understanding neuromuscular control of movement. IMU sensors capture precise kinematic data, crucial for tracking the mechanical aspects of limb and body movements. Stretch sensors measure joint angles, providing critical insights into joint kinematics and overall movement patterns. The higher accuracy and lower variance in predictions from multimodal data sources indicate that the iP3T model effectively utilizes the complementary information provided by each sensor type.

### 3.3 Attention scope for the iP3T

**Figure 8:**
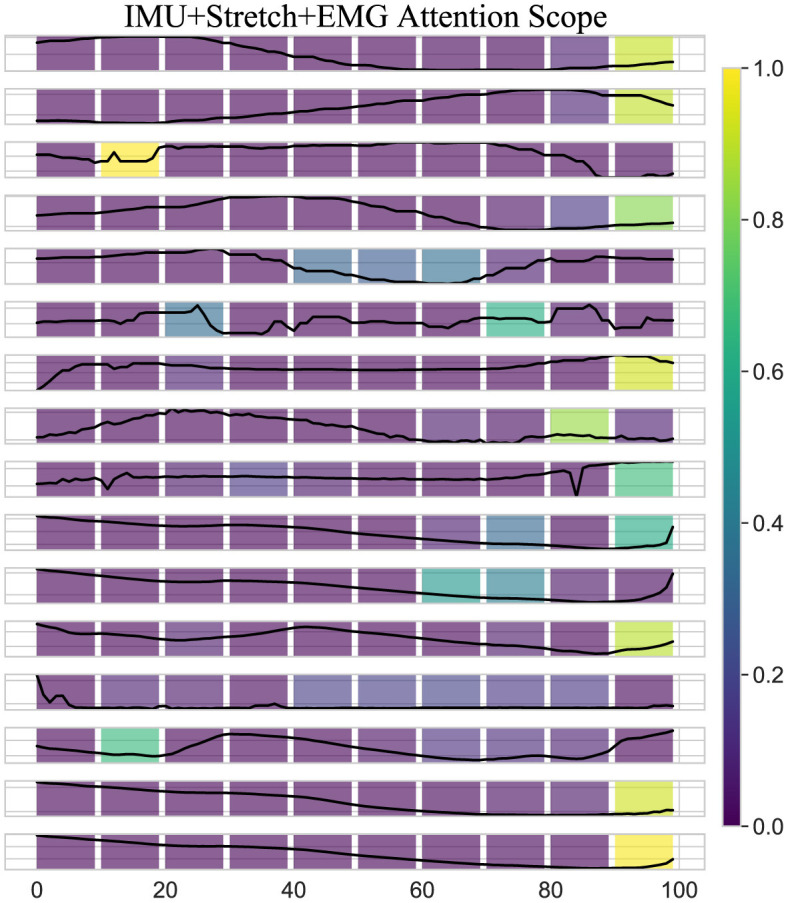
Attention scope visualization shows the attention scope of the iP3T model using IMU+Stretch+EMG data, highlighting the average attention values from the last six heads of the final three layers for a specific sample. The color gradient from purple to yellow represents increasing attention weights, indicating the importance of data points in predicting the current gait phase.

As shown in Figure 8, the attention scope visualization of the iP3T model using IMU, Stretch, and EMG data sources reveals crucial insights into how the model prioritizes different segments of time-series data during gait phase prediction. The attention mechanism assigns varying importance to different parts of the input sequence, with brighter areas on the heatmap indicating higher attention weights.

A key observation is the bright spots on the right side of the heatmap. These areas highlight the model's focus on the most recent data points when predicting the current gait phase, which intuitively makes sense as these points are likely to be most directly correlated with the current state of the gait cycle. The high attention weights here suggest that the model relies heavily on the latest sensor readings for accurate predictions, which is critical for real-time applications such as gait phase prediction in wearable exoskeletons. Additionally, bright spots at certain peaks earlier in the sequence indicate that the model also considers specific historical data points that may represent critical events or transitions within the gait cycle. These peaks likely correspond to key phases such as heel strike, toe-off, or mid-stance, where significant biomechanical changes occur. By assigning higher attention weights to these points, the model effectively integrates temporal context, understanding how past movements influence the current gait phase.

Moreover, the use of multimodal data sources like IMU, Stretch, and EMG enhances the model's ability to capture a comprehensive picture of the gait cycle. Each sensor type contributes unique information IMUs provide detailed kinematic data, stretch sensors offer insights into joint angles and movements, and EMG sensors capture muscle activation patterns. The attention mechanism leverages these complementary data streams, assigning higher weights where each sensor type offers critical information. The iP3T model effectively uses both recent and historical data points, focusing on key events within the gait cycle to make accurate predictions. This multimodal approach enhances the model's capability to integrate diverse and complementary information from different sensor types, achieving high accuracy and robustness in gait phase prediction, which ultimately benefits applications in wearable exoskeletons and other assistive technologies.

### 3.4 Comparison of various models with iP3T using different data sources

Table 3 lists the performance metrics (RMSE and R2) of different models in predicting gait phases using the IMU+Stretch+EMG data source, comparing CNN, GRU, LSTM, CNN+GRU, and CNN+LSTM with the iP3T model across four gait phases: pre-stance, post-stance, pre-swing, and post-swing.

**Table 3:** Metrics performance of different models on IMU+Stretch+EMG.

**Gait phase**	**Pre-stance**		**Post-stance**		**Pre-swing**		**Post-swing**	
**Model**	**RMSE**	**R2**	**RMSE**	**R2**	**RMSE**	**R2**	**RMSE**	**R2**
CNN	14.786	-1.911	3.194	0.871	2.577	0.793	18.715	-11.404
GRU	15.122	-2.044	2.872	0.896	3.823	0.544	20.382	-13.712
LSTM	14.936	-1.97	2.341	0.931	2.535	0.8	21.046	-14.686
CNN+GRU	9.439	-0.186	2.538	0.918	3.235	0.673	26.391	-23.664
CNN+LSTM	10.304	-0.414	1.786	0.96	2.289	0.837	14.107	-6.047
iP3T	**4.612**	**0.717**	**1.073**	**0.985**	**1.108**	**0.962**	**5.848**	**-0.211**

The bolded values represent the highest-performing metrics in the tables.

In the pre-stance phase, the iP3T model had an RMSE of 4.612, significantly lower than the next best model, CNN+GRU, which had an RMSE of 9.439. The iP3T model also showed a positive R2 value (0.717), indicating better fit, while all other models had negative R2 values, indicating poorer performance. In the post-stance phase, the iP3T model had an RMSE of 1.073 and an R2 of 0.985, outperforming all other models. The next best RMSE was 1.786 from the CNN+LSTM model, but its R2 was still lower than that of the iP3T. In the pre-swing phase, the iP3T model maintained excellent performance with an RMSE of 1.108 and an R2 of 0.962. The second-best model, CNN+LSTM, had an RMSE of 2.289 and an R2 of 0.837, indicating the robustness and accuracy of iP3T in this phase as well. In the post-swing phase, the iP3T model continued to lead with an RMSE of 5.848, while all other models had significantly higher RMSE values, such as 21.046 for LSTM and 18.715 for CNN. The iP3T model's R2 value was -0.211, which, although negative, was still better than the substantially negative R2 values of the other models.

The iP3T model consistently showed lower RMSE and higher (or less negative) R2 values across all gait phases, highlighting its superior performance in predicting gait phases using the IMU+Stretch+EMG data source. This performance can be attributed to iP3T's attention mechanism, which prioritizes the most relevant segments of the time-series data. As visualized, the model focuses mainly on the most recent data points, crucial for real-time gait phase prediction. Additionally, the attention mechanism identifies and emphasizes key historical data points representing critical gait events. In summary, compared to other traditional time-series models, the advanced architecture of the iP3T model and its effective utilization of multi-modal sensor data significantly enhance gait phase prediction performance. This makes iP3T highly suitable for applications in wearable exoskeletons and other assistive technologies, providing more accurate and reliable gait phase prediction.

### 3.5 The exoskeleton K5 metabolic cost measurement

**Figure 9:**
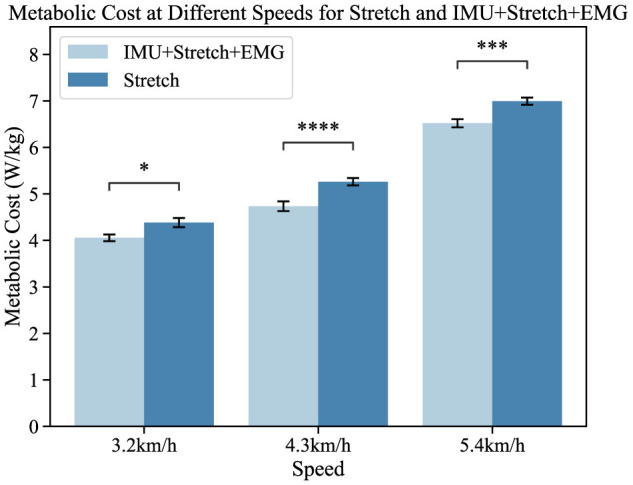
Metabolic cost at different speeds for the Stretch and IMU+Stretch+EMG conditions is shown. Light blue bars represent the IMU+Stretch+EMG condition, while dark blue bars represent the stretch condition. Metabolic costs were measured at speeds of 3.2, 4.3, and 5.4 km/h. *P*-value significance levels are denoted as follows: ^****^p < 1*e* − 4, ^***^p < 1*e* − 3, ^**^p < 1*e* − 2, ^*^p < 0.05, and ns, not significant.

As shown in Figure 9, the left bar chart represents the K5 oxygen consumption results from the iP3T model trained on IMU+Stretch+EMG data, while the right bar chart shows the results from the model trained on Stretch data. The mean values indicate that the gait cycle assistance guided by the iP3T model trained on IMU+Stretch+EMG data is significantly lower than that guided by the model trained on Stretch data. By providing external support or reducing leg burden, the assistance system can lower metabolic cost, as evidenced by reduced oxygen consumption.

In our study, participants showed no significant statistical differences. Compared to the single-modal Stretch data, the iP3T model trained on multimodal data significantly reduced metabolic cost: at 3.2 km/h, the cost decreased by 5.2% (*p* < 0.05); at 4.3 km/h, it decreased by 9.8% (*p* < 1*e* − 4); and at 5.4 km/h, it decreased by 6.1% (*p* < 1*e* − 3). The assistance system alleviates the burden on lower limb muscles, reduces muscle fatigue, and stabilizes the gait cycle. The positive impact of the assistance system on the gait cycle is evident, and the iP3T model effectively predicts gait cycle changes on the assisted treadmill. These findings provide significant insights for further optimizing the design of assistance systems and the application of the iP3T model.

## 4 Conclusion

In this study, we focused on developing and applying the iP3T model, emphasizing its interpretability, multimodal time-series data fusion, and exceptional performance in predicting gait phases. The iP3T model represents a significant advancement in wearable exoskeleton technology, specifically designed to assist and enhance gait for individuals with mobility impairments. By integrating data from multiple sensor modalities, including stretch sensors, IMUs, and sEMG, the iP3T model captures a comprehensive range of biomechanical and neuromuscular signals, providing a more detailed and accurate prediction of gait phases. Leveraging the strengths of each sensor type, the iP3T model achieves a level of precision and reliability that surpasses traditional single-modality approaches.

A key feature of the iP3T model is its interpretability, facilitated by attention mechanisms that assign varying levels of importance to different segments of the input time-series data. This capability allows us to understand which data points are most influential in the model's predictions. Visualizations of the attention weights reveal that the model prioritizes recent data points, which are most directly relevant to the current gait phase, while also considering critical historical data points that signify key gait events. This not only enhances the model's accuracy but also provides insights into the underlying biomechanical processes of gait, enabling more targeted and effective interventions.

Our experiments demonstrate the iP3T model's superior performance across various gait phases compared to traditional time-series models such as CNN, GRU, LSTM, and their combinations. The iP3T model consistently shows lower RMSE and higher R2 values, indicating better fit and prediction accuracy. These results highlight the model's robustness and its ability to effectively utilize the complementary information provided by the multimodal sensor data.

In practical applications, the iP3T model was tested on an assistive treadmill setup equipped with a flexible exoskeleton. The results showed that using the iP3T model for predicting gait phases significantly improved the effectiveness of the assistive system, reducing metabolic cost and enhancing stability. This demonstrates the potential of the iP3T model to provide real-time, accurate assistance, thereby improving mobility and quality of life for individuals with gait impairments.

The study also highlighted the critical role of each sensor type in the multimodal setup. For instance, the IMU sensors provided detailed kinematic data, the stretch sensors captured joint angle changes, and the sEMG sensors recorded muscle activation patterns. The fusion of these diverse data streams allowed the iP3T model to form a holistic understanding of the gait cycle, which is crucial for precise and adaptive assistance.

## Data Availability

The raw data supporting the conclusions of this article will be made available by the authors, without undue reservation.
